# Draft Genome Sequence of Microbacterium esteraromaticum MM1, a Bacterium That Hydrolyzes the Organophosphorus Pesticide Fenamiphos, Isolated from Golf Course Soil

**DOI:** 10.1128/MRA.00862-18

**Published:** 2018-08-02

**Authors:** Logeshwaran Panneerselvan, Kannan Krishnan, Suresh R. Subashchandrabose, Ravi Naidu, Mallavarapu Megharaj

**Affiliations:** a Global Centre for Environmental Remediation, Faculty of Science, The University of Newcastle, Callaghan, New South Wales, Australia; b Cooperative Research Centre for Contamination Assessment and Remediation of the Environment, The University of Newcastle, Callaghan, New South Wales, Australia; Georgia Institute of Technology

## Abstract

In this study, we report the first draft genome sequence of Microbacterium esteraromaticum MM1, isolated from golf course soil in South Australia. The genome possesses genes for the hydrolysis of organophosphorus (OP) pesticides and polycyclic aromatic hydrocarbon (PAH) degradation.

## ANNOUNCEMENT

The genus Microbacterium belongs to the family Microbacteriaceae, which has more than 96 species. Microbacterium esteraromaticum MM1 is a Gram-positive, aerobic, catalase-positive bacterium isolated from golf course soil in South Australia (1). The bacterium can degrade fenamiphos, an organophosphorus (OP) pesticide used to control nematodes in agricultural and horticultural crops and golf greens. The bacterium can hydrolyze fenamiphos and its oxidation products, namely, fenamiphos sulfoxide and fenamiphos sulfone, to their corresponding phenols. In the environment, fenamiphos undergoes oxidation to fenamiphos sulfoxide (FSO) and fenamiphos sulfone (FSO_2_), with both products having activity and toxicity similar to those of the parent compound (2, 3). The half-life of fenamiphos in groundwater under anaerobic conditions may even be more than 1,000 years (4). Additionally, fenamiphos and its oxidation products are highly toxic to aquatic and terrestrial organisms, with reported mortalities in birds and fish (5). Biological remediation methods using microbes or their enzymes are considered economical and ecofriendly for detoxification of pesticides. Microbes capable of degrading OP pesticides by hydrolysing P-O-C bonds have been previously isolated and characterized (6, 7). However, there are only very few reports available on the hydrolysis of fenamiphos and its oxidation products by axenic microbial cultures. Previously, Brevibacterium sp. MM1, a pure culture isolated from soil, demonstrated its ability to hydrolyze fenamiphos and its oxides in soil and water (8). Earlier, we reported (1) a nonpathogenic soil bacterium, namely Microbacterium esteraromaticum MM1, with exceptional ability to hydrolyze fenamiphos and its oxidation products to corresponding phenols.

The mid-log-phase culture grown in 6% tryptic soy broth (pH 6.0) was used for genomic DNA extraction. The genomic DNA was extracted with an UltraClean microbial DNA isolation kit (Qiagen, Australia) according to the manufacturer’s instructions. The integrity of the genomic DNA was evaluated by electrophoresis on 1% agarose gel. Whole-genome sequencing was carried out using an Illumina HiSeq 2000 next-generation sequencing platform provided by the Kinghorn Centre for Clinical Genomics at the Garvan Institute of Medical Research (Darlinghurst, New South Wales, Australia). *De novo* assembly was performed in the SPAdes assembler (9) version 3.9.0. The assembly yielded 225 contigs (>500 bp) covering a total of 3,625,508 bp, with an *N*_50_ value of 144,893 bp and a G+C content of 63.6%. Four contigs were shorter than 500 bp and were removed from the analysis. Annotations of contigs were carried out using the NCBI Prokaryotic Genome Annotation Pipeline (PGAP) (https://www.ncbi.nlm.nih.gov/genomes/static/Pipeline.html), which identified 3,363 genes, 3,277 coding sequences, 229 pseudogenes, 8 rRNA operons (4 complete and 4 partial), and 75 tRNA genes.

The genome also was found to contain gene clusters coding enzymes that might be involved in the metabolism of organophosphorus, organosulfur, and aromatic compounds such as phosphopyruvate hydratase (EC 4.2.1.11), salicylate hydroxylase (EC 1.14.13.1), catechol 1,2-dioxygenase (EC 1.13.11.1), shikimate 5-dehydrogenase (EC 1.1.1.25), monoamine oxidase (EC 1.4.3.4), and gentisate 1,2-dioxygenase (EC 1.13.11.4). Whole-genome sequences from the genus Microbacterium were previously reported in M. testaceum StLB037 (10), M. testaceum KU313 (11), M. laevaniformans OR221 (12), M. yannicii (13), and M. nematophilum (14). However, this is the first report of a draft genome of the genus Microbacterium with the ability to degrade OP pesticides and PAHs. The genome of strain MM1 facilitates an understanding of its biodegradation potential and also of its usefulness in the field of environmental remediation.

### Data availability.

The draft genome sequence of Microbacterium esteraromaticum MM1 generated in this study has been deposited at DDBJ/ENA/GenBank under the accession number PDVO00000000. The version described in this paper is version PDVO02000000.
